# Determinants of quality, latency, and amount of Stack Overflow answers about recent Android APIs

**DOI:** 10.1371/journal.pone.0194139

**Published:** 2018-03-16

**Authors:** David Kavaler, Vladimir Filkov

**Affiliations:** Department of Computer Science, University of California at Davis, Davis, California, United States of America; University of Bari, ITALY

## Abstract

Stack Overflow is a popular crowdsourced question and answer website for programming-related issues. It is an invaluable resource for software developers; on average, questions posted there get answered in minutes to an hour. Questions about well established topics, *e.g.*, the coercion operator in C++, or the difference between canonical and class names in Java, get asked often in one form or another, and answered very quickly. On the other hand, questions on previously unseen or niche topics take a while to get a good answer. This is particularly the case with questions about current updates to or the introduction of new application programming interfaces (APIs). In a hyper-competitive online market, getting good answers to current programming questions sooner could increase the chances of an app getting released and used. So, can developers anyhow, *e.g.*, hasten the speed to good answers to questions about new APIs? Here, we empirically study Stack Overflow questions pertaining to new Android APIs and their associated answers. We contrast the interest in these questions, their answer quality, and timeliness of their answers to questions about old APIs. We find that Stack Overflow answerers in general prioritize with respect to currentness: questions about new APIs do get more answers, but good quality answers take longer. We also find that incentives in terms of question bounties, if used appropriately, can significantly shorten the time and increase answer quality. Interestingly, no operationalization of bounty amount shows significance in our models. In practice, our findings confirm the value of bounties in enhancing expert participation. In addition, they show that the Stack Overflow style of crowdsourcing, for all its glory in providing answers about established programming knowledge, is less effective with new API questions.

## Introduction

The social coding movement and the phenomenon of crowdsourcing have made eminently useful software development resources and services available at low cost. The Stack Overflow question and answer site and various Open Source Software forges, like GitHub, are transformative resources; they enable the creation, promulgation, and archiving of new knowledge and artifacts on an as-needed basis. They are often also very responsive: most questions on Stack Overflow are answered within minutes, and pull requests get reviewed, merged, and released into the codebase of large projects within days. In fact, Stack Overflow and Open Source work quite well as a coupled, interdependent system, with the former providing almost instantaneous documentation for the latter, and developers of the latter serving as askers and answerers in the former’s gift economy. This system is critically predicated on both a short turnaround time and the existence of enough knowledgeable users to provide needed expertise. As soon as one or both of those conditions are unmet, the programming and documentation resources get decoupled.

On Stack Overflow, users are accustomed to having their questions answered rapidly; according to Vasilescu *et al.*, the mean time to an answer is between 17 and 47 minutes, depending on subject area [1]. In addition, users want high quality answers, *i.e.*, answers that address the core of their question, which may additionally address related concerns not explicitly put forth by the original asker. It has been shown that Stack Overflow is effective for code reviews and conceptual questions [2], as well as providing adequate API coverage [3]. However, many questions are asked which don’t get fast enough attention from the crowd [3]. In addition, though work has been done to reduce the number of low-quality posts on Stack Overflow [4, 5], issues regarding answer quality and timeliness still remain for important classes of questions.

In particular, we noticed a stark delay in getting good answers to questions related to recently introduced Android APIs; or, “new APIs”. We define an API as being “new” if it has not been modified between the time of its introduction and the time the question mentioning it is asked. Our data shows that questions referencing new APIs are answered, on average, *8,000* minutes (about 5.5 days) slower than questions referencing only old APIs. That is a very large difference in practice, especially in the hyper-competitive markets of Android apps and modern software engineering automation technologies, like continuous deployment. How are questions and answers about new APIs different than those about old ones, in terms of length, quality, incentives, *etc.*.? And, more importantly, can question askers do anything to hasten adequate answers?

Motivated by the above, here we are interested primarily in identifying the effects of new API mentions in Stack Overflow questions on three outcomes of interest, which are important in practice: the time to a first good answer, the number of answers, and the answer quality. To study these outcomes, we fused data from two sources: Stack Overflow and the Google Play store. From the latter we gathered function invocation data on 20,014 Android apps, and from the former we gathered questions and answers that mention Android APIs used in those apps. We then built separate regression models for time to a first good answer, number of answers, and answer quality as functions of question attributes, bounty usage, and many confounding variables. Our findings show that:

- Questions involving new Android APIs attract more answers over the question’s lifetime compared to those questions involving older APIs.
- Questions involving new APIs receive good answers more slowly within the first 2 days. After 2 days, new APIs attract faster answers. Among questions answered after 2 days, only 46% are answered within 1 month.
- Adding a bounty not only reduces time to answer on average, but also flattens the long tail and increases density towards faster answers. Bountied questions also receive more answers. However, the exact reputation value of the bounty does not seem to matter.
- Answers that come during a bounty period are of higher quality. Questions referencing new APIs have no significant effect in receiving higher quality answers.

In what follows, we first discuss background and our research questions, followed by related work, data, methodology, results, discussion, threats to validity, and conclusions.

## Background and research questions

In this work we are interested in the popular Android ecosystem. Android is an open-source, Linux-based software system, used usually on mobile devices. It is built around the Android operating system (OS) developed and updated regularly by Google. The Android OS can be interfaced using the Dalvik virtual machine through Java-based application programming interfaces (APIs); more commonly called the Android API. Android gets updated by Google regularly with new features, which can be accessed through new versions of old APIs or completely new APIs. To provide backwards compatibility and to allow developers to specify what version(s) of the API their particular application targets, the Android API is split into levels, corresponding to API versions. For example, a device running Android version 4.0.3 can support up to API level 15, *i.e.*, it can run applications which target API levels from 1 − 15.

The Android framework presents a unique opportunity that other OSS app ecosystems do not: the availability to download many real applications. Namely, the Google Play store (https://play.google.com) has a multitude of free Android applications that are open for download. These applications can be simply converted into a reliable byte code format, revealing exactly which Android APIs are called by a given application (described further in Data) with high reliability.

Modern software developers use Stack Overflow and related social coding sites extensively [1, 6, 7]. Thus, it is important that Stack Overflow meets both the needs and expectations of its users—fast, high quality answers, with multiple perspectives (*i.e.*, more answers for a given question). Because of the popularity of mobile apps and the low cost of entry in the developer markets, Android use is growing among developers. Due to constant updates to the Android OS and the introduction of new APIs, Android questions on Stack Overflow are extremely popular and occupy a significant fraction of all questions there; as of this writing, Android is the 5th most popular topic. Fast, high quality answers, especially about new APIs, are thus important for productivity and to, *e.g.*, maintain relevancy of software with respect to current demand; else, applications may fall to the wayside.

The need for timely, quality answers is addressed on Stack Overflow via different mechanisms. *E.g.*, users can choose to place a *bounty* on a question after the question is 2 days old. A bounty is an extra reputation point bonus applied to a question, funded by the bounty creator’s own reputation score. The bounty creator can choose to spend between 50 and 500 reputation (in accordance to various rules) on a bounty. The ability to attach a bounty to a question requires some amount of participation in Stack Overflow (*i.e.*, a total of 75 reputation). The predominant function of the bounty system is to attract *extra attention* to a question. Questions with active bounties are put into a special “featured” section in the main Stack Overflow question list, granting them increased visibility.

The public availability of Google Play and Stack Overflow data enable us to link the introduction and use of APIs in Android apps (as revealed from their byte code) to questions asked about particular APIs on Stack Overflow. We use this linked resource to study the differences in answers about new, *vs.* old, APIs, and the role of bounty incentives.

### Research questions

We sought to model the differences in answer latency, quality, and quantity between Stack Overflow questions referencing new versus old APIs. New APIs, by definition, have no existing crowd documentation for users to rely upon. Specifically, there are fewer Stack Overflow questions or answers regarding the proper usage of new APIs than older APIs, on average. In addition, new APIs may not be as well documented, as their creators have not had the chance to receive feedback from general users to indicate aspects which require clarification. Further, there are fewer users knowledgeable about new APIs (as they are new), which may increase the proportion of slower or lower quality answers to such questions. In addition, we want to account for confounds that differentiate any two questions, like question quality, length, descriptiveness of the title, and similar characteristics which are visually apparent to readers.

**Research Question 1**: Given that an API referenced in a question is either a new one or an old one, which, if any, among a number of observable characteristics of the question are determinants of the answer quantity, quality, and latency?

Bounties can be seen as a layer on top of the old Stack Overflow knowledge exchange system which allows a user to “pay” with reputation points for additional services on top of the basic, public ones. Though research on the effects of the bounty exists, it is still unclear exactly what (if any) is the outcome of the bounty offering. Some potential outcomes are: increased quality of answers, reduced time until a quality answer, attracting people who can answer difficult questions better or faster, *etc.* But are any of these potential outcomes realized, when controlling for the effect of API newness?

**Research Question 2**: Are bounties associated with more, faster, or higher quality answers, when controlling for the presence of new APIs?

## Related work

Prior work related to this research falls mainly into three areas: the usefulness of Stack Overflow for software engineers as a development resource; work on Stack Overflow Q&A quality and latency; and work regarding the mechanisms provided by Stack Overflow in order to incentivize participation and thus increase answer count, speed, and quality.

### Stack Overflow as a development resource

Stack Overflow has been used as a subject of study by many researchers in software engineering. Treude *et al.* developed a taxonomy of question types, and categorized questions based on this taxonomy [2]. In addition, they discussed which types of questions receive more answers. Other researchers have discussed how developers use social media sites (such as Stack Overflow) as part of their normal workflow [6] to ask a wide variety of questions [8]; many tools have been developed to aid in this process [9, 10].

Much work exists that examines the usefulness of Stack Overflow as a resource to aid the development process. Parnin *et al.* found that when performing Google searches of the API methods in jQuery, 84.4% of API methods had a Stack Overflow post returned on the first page of the Google search [11]. In addition, highly used APIs are also generally discussed more [12]. But, many articles and questions get asked which don’t get fast enough attention from the crowd [3]. Although Jiau and Yang argue that more obscure questions benefit from a “trickle-down” effect from similar questions [13], some questions are more time-critical and may need an answer even faster.

### Stack Overflow Q&A quality and latency

Closely related to our work are studies on analyzing question and answer quality on Stack Overflow, and predicting the best answers for particular questions. Ponzanelli *et al.* examined the existing review queue system on Stack Overflow for automatically detected “low quality” posts, providing suggestions and alterations to reduce the queue size and increase its identification accuracy [4]. Dalip *et al.* used user feedback in order to provide suggestions as to how to reduce the number of low-quality posts on Stack Overflow [5].

Baltadzhieva and Chrupala surveyed various metrics from prior work in determining Stack Overflow question quality, including tags and terms within the question itself [14]. Tian *et al.* use answer acceptance as a proxy for measuring a “good” answer [15]. Similarly, Shah and Pomerantz examined the Yahoo! Answers data set and used human assessments through Amazon Mechanical Turk to build a model for predicting which answer would be chosen as best by the question asker [16]. In our analysis of the data, we found that very few answers are actually designated as “accepted”, even though the answer quality might in fact be quite high. In addition, as noted by Gantayat *et al.* [17], often the accepted answer is not the best according to community popular vote. Thus, using the accepted answer as an indicator of answer quality may not accomplish what is intended.

To study the effects of new APIs on answer quality, we require a method for labeling post quality; this comes down to answering the question: what makes a post “good”? In our work, we create post quality labels based on work by Ravi *et al.* [18]. Ravi *et al.* address issues of conflating quality with popularity, as a question that is viewed many times has more chances to get votes. Through theoretical arguments and some empirical analysis, they decide to consider the quantity *p*_*i*_ = *s*_*i*_/*v*_*i*_, where *s*_*i*_ is the score for question *q*_*i*_ and *v*_*i*_ is the view count. Here, the view count acts as a control for popularity. They go on to argue for labeling questions with *p*_*i*_ = 0 as “bad”, and labeling questions with *p*_*i*_ > 0.001 as “good”. We use this labeling strategy in our work.

Other researchers have studied the topic of answer speed [19–21] in community question and answer sites, with varying degrees of success using a variety of methodologies. Here, in contrast to most prior work, we are interested only in questions and answers related to Android—that can be linked to Android APIs—and use a standard regression framework for inference. Linares-Vásquez *et al.* [22] found that Android API behavior modifications trigger much discussion on Stack Overflow, indicating that there is interest within the community regarding new or changed Android APIs, meaning our restriction to studying only Android APIs should not be debilitating. However, we note that our focus on Android alone may affect generalizability to other domains.

### Incentivizing Stack Overflow users

Stack Overflow has implemented a number of incentive mechanisms to encourage user participation, including badges (which serve as rewards for achieving various feats), reputation (gained through participation), and various privileges awarded upon reaching reputation milestones. Reputation on Stack Overflow is gained through various methods, primarily by receiving up votes on questions and answers and by having an answer being marked as “accepted”, indicating that the asker “received an answer that worked for him or her personally” (https://stackoverflow.com/help/accepted-answer). These incentive mechanisms have proven to be effective in garnering activity and popularity [23, 24].

Movshovitz-Attias *et al.* found that high reputation users are the primary source of high quality answers [25]. Grant and Betts examined three specific Stack Overflow badges in detail, finding that users tend to increase their activity in order to attain these badges [26].

However, it has been noted that Stack Overflow’s incentive mechanisms can be at odds with question and answer quality. Jin *et al.* studied gamification-influenced member tendencies on Stack Overflow, arguing that the fastest response often “wins” the most reward [27]. Bosu *et al.* studied exactly what actions a user can take to build reputation quickly [28], concurring with Jin *et al.*, finding that a number of non-expertise related strategies can effectively increase reputation (*e.g.*, activity during off-peak hours). In addition, there have been discussions about declining quality due to the emergence of an “old boys’ club” mentality [29], and the existence of “one-day flies”; the vast majority of Stack Overflow users only post once [30]. Posnett *et al.* found evidence that users on Stack Exchange (the umbrella under which Stack Overflow lies) do not increase in answering expertise over time [31]. In light of this, it is important to understand how to attract attention to one’s questions in an effective manner and from the true experts. This is especially true for questions about novel topics that have only recently arisen, *e.g.*, new APIs.

Anderson *et al.* set out to predict the long-term value of a question, as well as whether a question has been sufficiently answered [32]. To accomplish the latter, they attempt to predict whether or not a question will attain a bounty, which serves as an indicator that the question was not yet adequately answered. Berger *et al.* studied bounties and their effect on question performance compared to non-bountied questions [33]. Though these works use the bounty as a measure of existing answer quality, they do so in a different way than us, and to a different end. Here, we are interested in determining whether or not the bounty system *improves* answer quality, number of answers, or response time, controlling for the effect of new APIs in a question.

## Data

In the following subsections, we describe our data and how it was collected, our strategy for identifying and linking APIs to Stack Overflow questions, various statistics we calculated for use in our models, and how we filtered our data to ensure model robustness.

### Data collection

Stack Exchange provides public data dumps periodically for all the sites within the Stack Exchange network, including Stack Overflow. We use data from the Stack Overflow data dump dated March 16, 2015 (retrieved from https://archive.org/details/stackexchange). From this data, we extracted a rich set of variables, including question view count, user-defined question tags, question and answer scores, and question asker and answerer reputations. In addition, we calculated a large set of variables based on this data including number of words in the body of a post, amount of code in a post, question title length, and question asker and answerer “wisdom” scores (explained below). A full list of collected variables used in our models can be found in Table 1. Note that Table 1 also contains data gathered from other sources, described below.

**Table 1:** Model variable descriptions.

Variable name	Description
F.QQualityLabelGood	Label for question quality.
TimeToBounty	Time to bounty start (days). Equal to 0 for questions that never receive a bounty.
TimeToAnswerMins	Time to answer, in minutes.
QCreationDate	Number of days between the first Stack Overflow post (ever) and the question creation date.
QOwnerNQ	Total number of questions created by the question owner.
Q/AOwnerReputation	Reputation for the post owner.
QOwnerAge	Number of days between question owner’s account creation and the question creation date.
Q/AMEC	Mean Expertise Contribution (MEC) for the post owner.
QTitleLength	Title length for the question.
QNTags	Number of tags for the question.
Q/ABodyNWords	Number of words in the post body, not including code.
Q/ABodyCharsOfCode	Number of characters of code in the post body, including both code blocks and inline code segments.
Q/ANSwitches	Number of structural changes in the post body.
Q/ABodyURLCount	Number of URLs in the post body.
QNComments	Number of comments for the question.
QNeed	Calculated question documentation need, scaled.
F.Bounty	A factor indicating whether or not the first good answer was provided during a bounty period.
F.Added	A factor indicating whether or not the question references a newly added API.
APIDiffTime	Minimum number of days from which a linked API was changed for all linked APIs in the question. If a new API is present, this is the number of days since the new API was added.

All numeric explanatory variables are logged, except APIDiffTime, TimeToBounty, and QNeed.

In addition to Stack Overflow related data, we developed and used a metric that requires API call counts from real Android applications. To serve this purpose, we wrote a custom crawler to download free applications from the official Google Play app store (https://play.google.com). The crawler operates by “clicking” each link on the front-page of the Google Play store, recursively “clicking” links on successive pages until all links have been exhausted. Note that this search is not entirely random, but attempts to emulate a random search through the space of applications. This pseudo-random search is necessary as there is no simple method of extracting a random application from the Google Play store. This crawler downloaded a total of 20,014 applications. We then converted the apps to a more human-readable byte code format using APKTool [34]. We processed the extracted byte code files by counting function invocations (invoke-virtual, invoke-super,invoke-direct,invoke-static and invoke-interface).

We also gathered documentation data from Android source code by running *Javadoc* with a custom Doclet [35]. This allowed us to gather data such as class documentation line counts, number of inner classes (*e.g., Animator.AnimatorListener*), and average method documentation lines.

### Android change data

To collect API change data, we use the official Android change lists provided by the Android SDK manager. However, some of these change lists are incomplete. For example, according to the documentation website, the class *android.accounts.AccountManager* was added in API level 5. However, the change list packaged with the SDK release has no mention of this class (change list can be viewed here: https://goo.gl/I4tsPl). For APIs with this issue, we assume that the API was added in API level 1. In this work, **we identify an API as “new” if it has not been modified between the time of its introduction and the time the question is asked**. We note that it is likely clearer to classify new APIs as those that were added only in the most recent framework change. However, there are a number of reasons we do not define new APIs in this manner. Developer adoption of new frameworks can be relatively slow for existing applications, as updating to the newest framework versions may involve risk. Although the Android framework claims strict backwards compatibility for their APIs, and rarely remove APIs outright, there is always an inherent risk of breaking the current code base with any underlying framework update. In addition, there are periods of time in which new Android frameworks are released very rapidly; for example, API levels 2 − 7 all released within the same year. If we define new APIs as those newly introduced in the latest update, we are severely limiting our data for a number of time points, as there is very little time for new APIs to be discussed. Thus, in order to have enough data to reliably model, we define a new API as described in bold above.

### Stack Overflow question API links

**Fig 1:**
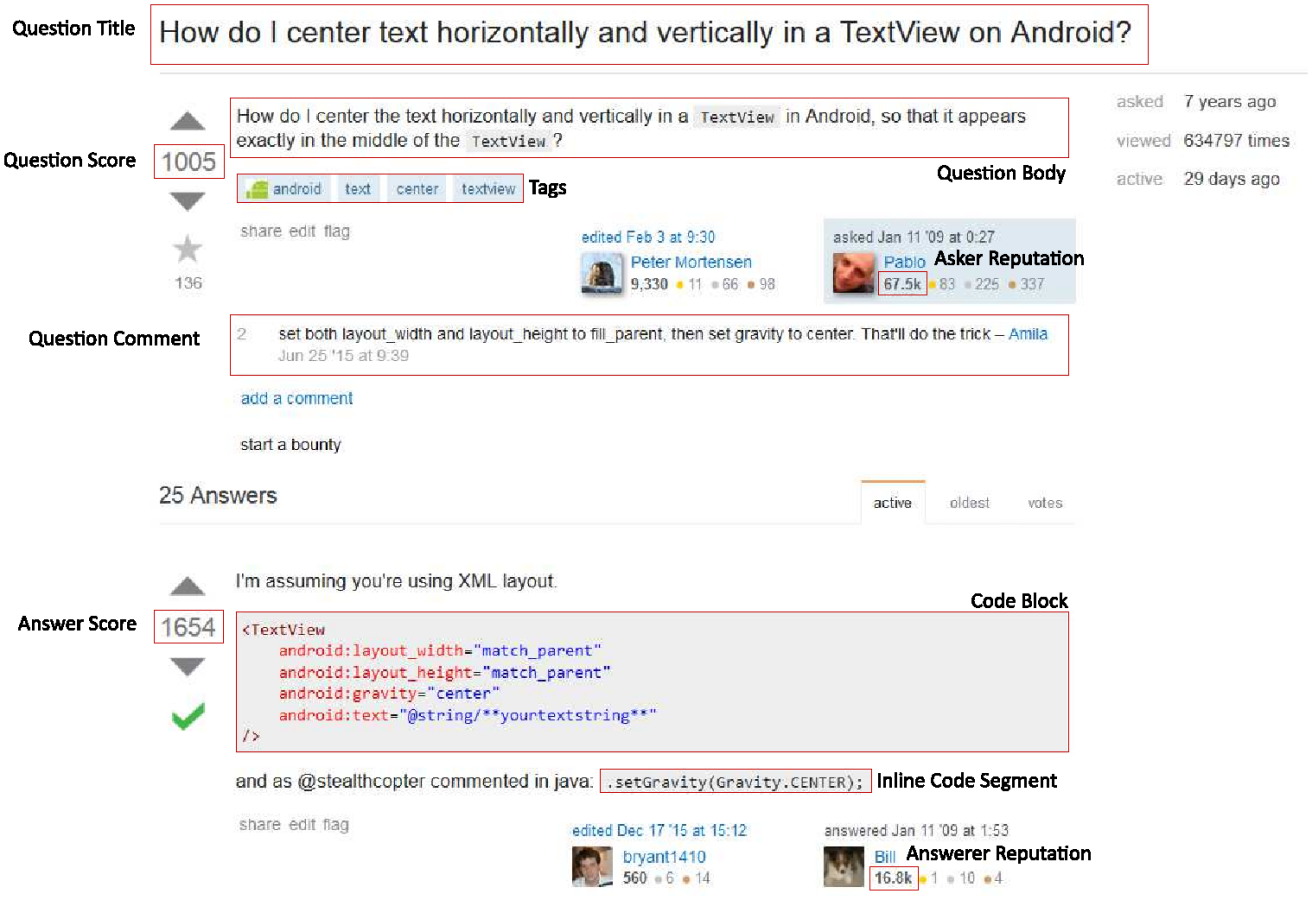
A question and answer on Stack Overflow. Some relevant variables are outlined in red.

To identify questions discussing relevant APIs, we examine the body of Stack Overflow questions to extract *links* to APIs. The link types considered here are:

1. *Tag links*: A class name match occurring in the tags section of a Stack Overflow question.
2. *Href markup links*: A class name match enclosed by HTML <a></a> tags, referring back to the Android documentation site.
3. *Title links*: A class name match occurring in the title of the Stack Overflow question.
4. *Code links*: A class name match *exactly* occurring within HTML <code></code> segments—this means large code blocks (*i.e.*, those contained within <pre><code>…</code></pre> tags) are not considered when identifying API links (Fig 1, “inline code segment”). Large code blocks were not considered in API linking as they create large numbers of false positive links when users post long code segments to show how they have tried to solve their problem in question.

This is a similar strategy as that used in prior work [3, 12], with some alterations. These alterations were made to focus on identifying *true positive* links, while minimizing *false positives*. For our models to be useful in answering our research questions, we believe it is more important to make sure our data set includes only properly linked APIs than to cover all questions referencing APIs; hence the emphasis on true positives. In addition, we consider an API as a particular *class mention*, *e.g.*, *android.app.Activity*, rather than by *method mention*, *e.g.*, *android.app.Activity.onCreate()*. This is due to the fact that method names are often more generic than class names, *e.g.*, a method named *start()* may belong to many classes. When determining links, searches for both fully qualified class names (*e.g.*, *android.app.Activity*) were considered along with class names alone (*e.g.*, *Activity*).

We note that there are other approaches to extract API links from Stack Overflow posts, as done by Rigby and Robillard [36]. However, we could not find an existing open implementation of their tool to apply to our work. In addition, their work relies on an island parser; open and usable island parsers for general code are difficult to come by and, by our experience, often prone to error (due to the difficulty of the island parsing task). Thus, due to the lack of reliable open implementations, we use the strategy as outlined above.

In order to provide an estimate for the precision and recall of our linking strategy, we performed two manual case studies on separate sets of 50 randomly selected questions. The first set consists of questions that were detected by our linking strategy, used to estimate precision. This set was also used for our case study on new API interest in Stack Overflow, described in a later section of this work. Among this set, we report an estimated precision of 96% (48/50).

The second set consists of questions manually identified by the authors of this work as being “explicitly about” or “involving” an API (the criteria for these classifications are described in the aforementioned manual case study, described below), used to estimate recall. We then ran our linking strategy on these questions, which correctly identified 30 links; a recall of 60% (30/50). This relatively low recall is expected, as we specifically designed our linking strategy to emphasize precision (true positives), as described above, knowing this would affect our recall.

### Combining documentation, API linked posts, and usage data

As described above, we have data from a multitude of sources: Stack Overflow questions and answers linked to APIs, documentation metrics for each Android API level, and function invocations in real applications. To combine these data sources for use in our models, we performed a number of steps.

As our models are at the post level (*i.e.*, question or answer), we must aggregate data per post. For documentation metrics, *e.g.*, number of documentation lines for a linked class, we discover the most recently released API level given the post’s creation date, and attach the corresponding documentation metric. In contrast, for function invocation data, we do not discern between API levels. We note that a more accurate approach would be to aggregate function call counts for each application’s target API level, and attach that data to the linked post’s discovered API level (*i.e.*, the latest API level as of posting). However, this would require us to gather applications that target many more API levels. As the Google Play store does not give access to older versions of applications, we do not have enough data to discern between API levels for function invocations.

In summary, we calculate documentation metrics per API level, and aggregate function invocation counts across all API levels. We then attach these metrics to a given post based on the post’s linked API. All other variables used in our models (as described in following subsections) are calculated at the post level (excluding residual question need, which uses the same combining methodology described above).

### Wisdom scores

As we are primarily interested in assessing the effect of new APIs on various outcomes (*e.g.*, response time), we must control for asker answerer expertise, which can also affect our outcomes of interest. Thus, we require a metric to measure expertise within the framework of Stack Overflow *i.e.*, not necessarily purely technical expertise. There has been much interest in measuring user expertise on Stack Overflow, with researchers investigating multiple dimensions that contribute to expertise, along with applications of measures [37–40]. However, most definitions of expertise are coarse-grained; *e.g.*, merely using reputation, or some simple function of reputation. Here, we leverage work by Yang *et al.* [41]. They introduce a novel metric called Mean Expertise Contribution (MEC), referred to as a “wisdom” score. In essence, this metric considers two dimensions of user wisdom or expertise: the *debatableness* of a question, and the *utility* of an answer. MEC is defined as:

$$\begin{array}{c} {\text{MEC}}_{u,t}=\frac{1}{|Q_{t}^{u}|}\sum_{\forall q_{i}\in Q_{t}^{u}}\text{AU}\left(u,q_{i}\right)*\frac{D(q_{i})}{D_{t}^{avg}} \end{array}$$

where:

- $Q_{t}^{u}$ is the set of questions from user *u* on topic *t*. In this work, we consider only one topic: Android (as defined by Stack Overflow question tags).
- $\text{AU}(u,q_{i})$ is the *utility* of the answer provided by user *u* to question *q*_*i*_; $\text{AU}\left(u,q_{i}\right)=\frac{1}{Rank(a_{q_{i}})}$ *i.e.* the inverse rank of the answer provided by *u* for question *q*_*i*_. A rank of 1 indicates the highest scoring answer for a question post. Thus, a larger AU indicates a higher expertise level shown by user *u* for question *q*_*i*_.
- $D(q_{i})$ is the *debatableness* of question *q*_*i*_, calculated as the number of answers |*A*_*q*_*i*__| provided for question *q*_*i*_.
- $D_{t}^{avg}$ is the *average debatableness* of all questions related to topic *t*, calculated as $\frac{1}{|Q_{t}|}*\sum_{\forall q_{j}\in Q_{t}}\left|A_{q_{j}}\right|$

A value of *MEC*_*u*,*t*_ = 1 indicates that user *u*, on average, provides the best answer to averagely debated questions. *MEC*_*u*,*t*_ = 0.5 indicates that user *u* ranks second in answering averagely debated questions, or ranks first in answering less debated questions. We use this metric in our models.

We acknowledge that the MEC metric can summarize multiple phenomena with the same value, which may be initially seen as a drawback. However, we chose this metric precisely because of its summarizing capacity; specifically, its ability to balance the influence of both user activity and contribution quality. As described by Wierzbicki *et al.* [42], identifying expertise in community question and answering (CQA) systems is difficult. Thus, novel approaches are necessary as, *e.g.*, existing approaches (other than MEC described here) often conflate activity with expertise. Though we acknowledge that there are potential drawbacks of this metric, this metric is considered a state-of-the-art approach for measuring user expertise.

### Text-based variables

The Stack Overflow data dump includes the body of all posts including HTML markup as displayed on the website. Using this data, we can extract variables in addition to API links, including the number of words in a post, the amount of code in a post, and specific information about the structure of the HTML used in the post.

To extract the amount of code in a post, we take care to differentiate between code blocks and inline code segments, as shown in Fig 1. These two types have slightly different HTML markup on Stack Overflow. We calculate both the lines of code and total characters of code in both code blocks and inline code segments.

To extract word-based variables, we use JSoup (http://jsoup.org/) to remove code *blocks* and send the resulting raw text (*i.e.*, without HTML tags) to the Stanford CoreNLP library [43] to tokenize and detect sentences. This way, our word-based variables include inline code *segments*, but not code *blocks*. This is because inline code segments are often used as part of a natural language sentence, and we believe they should be treated as words. On the other hand, code blocks are purely formatted code, which should not be analyzed as natural language text. Prior work also shows that natural language text is just as important as code in a Stack Overflow question [44], thus we must have some representation of language in our models.

Posters will often include links to documentation and related Stack Overflow questions and answers. Thus, we extract the number of URLs in the body of the post, the number of user-defined tags for the associated question, and the length of the title of the associated question.

Finally, we calculate the number of switches between HTML tag types in the base-level body of a post. This is a measure of *structural complexity*. We theorize that the more switches between natural language text and code in a post, the more complex the post is in terms of content. In addition, we believe that some structural information should be included in the models as more structure can increase readability in terms of visual clarity. To calculate this, we extract HTML tag sequences and count the number of switches between *different* tag types at the base-level. For example, if we see a sequence of tags such as:

<a>…</a>

<p>…</p>

<p>…</p>

<code>…</code>

<pre>

      <code>…</code>

</pre>

we would count three structural switches: one switch from <a></a> to <p></p>, one switch from the second <p></p> to <code></code>, and one switch from <code></code> to <pre><code></code></pre>. The transition between the two <p> tags is not counted as a switch, as these are the same tag type. Note that there is an embedded <code> tag within the <pre> tag—as this is not at the base-level of the post body, we do not count this as a switch. This also avoids double counting code blocks which are visually a single unit, but could be considered two structural units if one does not count the HTML tags in the aforementioned way. In Fig 1, the number of switches for the answer would be equal to 2, even though there is an inline code segment in the final paragraph.

### Question and answer quality

As noted in Related work, our method of classifying question and answer quality is drawn from work by Ravi *et al.* [18]. As stated, they define the quantity *p*_*i*_ = *s*_*i*_/*v*_*i*_, where *s*_*i*_ is the score for question *q*_*i*_ and *v*_*i*_ is the view count; the normalization by the view count acts as a control for popularity. They then argue for labeling questions with *p*_*i*_ = 0 as “bad”, and labeling questions with *p*_*i*_ > 0.001 as “good”.

We argue that similarly for questions, answer quality should be a function of associated view count and answer score. However, for answers, the use of view count is slightly different as we only have access to view count at a question-level. Thus, it is likely that some answers are viewed more than others, and that the view count variable does not accurately reflect this. As a result, in all relevant models we control for the time difference in question creation to answer creation. This serves as a control to alleviate the bias that the view count variable has towards answers that are created earlier.

Note that it may appear that a post decays in quality (*p*_*i*_) over time, as views continue to increase in time. However, although more people view the post in time (increasing *v*_*i*_; decreasing *p*_*i*_), these people also can up-vote the post (increasing *s*_*i*_). If we assume that people are equally likely to up-vote a post across time, then *p*_*i*_ is still valid; we believe this assumption holds true in practice, though there is no prior work on exactly this phenomenon. We acknowledge that this underlying assumption may not be true, and thus poses a threat to validity.

### Residual question need for documentation

In previous work [12], we addressed the idea of Stack Overflow as a documentation source, and built a model to predict the number of API linked Stack Overflow questions using actual API usage in free Android applications and a number of controls. The model is of the form:

$$\begin{array}{ccc} & & \text{Number}\;\text{of}\;\text{API}\;\text{linked}\;\text{questions}=\beta_{0}+\\ & & \;\beta_{1}\text{Number}\;\text{of}\;\text{API}\;\text{calls}\;\text{in}\;\text{free}\;\text{apps}+\beta_{2}\text{Source}\;\text{documentation}\;\text{lines}\;+\\ & & \;\beta_{3}\text{Number}\;\text{of}\;\text{inner}\;\text{classes}\;+\beta_{4}\text{Class}\;\text{documentation}\;\text{lines}\;+\\ & & \;\beta_{5}\text{Average}\;\text{method}\;\text{documentation}\;\text{lines}\;\text{per}\;\text{class} \end{array}$$

where the *β*_*i*_ are estimated model coefficients, fit on a sample of real Android applications and associated documentation, combined in the same manner as described in this work above. These variables were chosen through model selection and identification based on hypotheses outlined in the mentioned work; we refer the reader there for more in-depth information as to how these variables were selected and modeled.

As this model predicts the number of linked Stack Overflow questions per API, where questions correspond to documentation, we view the **negative** of the residuals of this model as representing *documentation need* (residual = observed value—predicted value). If the negative residual of the documentation need model is negative, our model predicts a lower amount of documentation than exists on Stack Overflow, indicating that the API is *over-documented*; if the negative residual is positive, the API is *under-documented*. We emphasize that the idea of using this metric is to represent API documentation need as a function of API usage in real applications, and a number of controls. The theory behind this is that, generally speaking, with more knowledge seekers there is an increased probability of nuanced, specific questions, as the general usage questions have already been answered and thus are less likely to turn up again. As a result, an API that is used more will likely require more documentation to satisfy users’ needs than one that is used less. This also provides another metric for the *currentness* of Stack Overflow. If Stack Overflow is very current, *i.e.* up-to-date in terms of API documentation, then documentation need for APIs will generally be low. This metric is taken into consideration along with analysis of new APIs to measure currentness. We acknowledge that the theory above is one possible explanation of many for this phenomenon. However, our definition of documentation need comes directly from the definition of the residuals; if one believes that the model outcome represents documentation, then the residual represents documentation need.

### Data filtering

Posts that are older than one year that meet a certain set of criteria are deleted from Stack Overflow and the underlying data dump [45]. Note that the data we use and the resulting metrics calculated based on the data are from the *snapshot date*, *e.g.*, reputation for users is calculated as of the date of the snapshot, not the date of the posting. This is due to the way that Stack Overflow structures its data dump. To address this, we only consider questions and answers created before March 16, 2014 (1 year prior to the dump date) to avoid issues of sample bias in our models. Note that this should not significantly affect the interpretation of reputation, which we use as of the snapshot date. The point of only considering posts that were created before March 16, 2014 is to avoid posts that may be soon deleted. Automatically deleted posts have low score by definition, and also cause very small changes to reputation (if any), due to the criteria by which posts are deleted. Our models aggregate over many individuals and questions and answers, so small reputation discrepancies should not matter in the aggregate.

We primarily use two supplied tables from the Stack Overflow data dump: the Posts and Votes tables. The Posts table contains the posts themselves along with meta-information. The Votes table contains each vote (*e.g.*, up, down, flagged as inappropriate, *etc.*) for each post. There are a number of consistency issues with these two tables that must be addressed before they are used in our models.

Posts which are deleted are not contained in the dumped Posts table. However, votes for these posts are sometimes not deleted from the Votes table. Additionally, if a post is migrated from Stack Overflow to somewhere else in the Stack Exchange network and a bounty was started while the post was still on Stack Overflow, the Votes table will contain an entry for the start of the bounty while it will not contain an entry for the end of the bounty. As a result, we only look at question threads which have not been migrated or deleted as of the data dump. There are a number of observed discrepancies in the Stack Overflow data set, mostly arising due to deleted posts, migrated posts, and related administrative actions. We made a best-effort attempt to clean the data of these inconsistencies. These specific issues affect a vast minority of our data points (<1%) and should have a negligible effect on our outcomes.

For our models, we do not consider answers from users who have deleted their accounts or answered without an account, as this causes their reputation scores to be lost in the data. Similarly, we do not consider questions in which the question asker has deleted their account or asked without an account. After filtering for all of these issues, our data set reduces from 633,659 Android-tagged questions to 410,287 questions. The final step in filtering is to consider only those questions which are positively linked to an API, leaving us with 22,366 questions for all models presented.

## Methodology

To answer each of our research questions, we have separate models using various forms of linear regression. This allows us to inspect the relationship between our response (*dependent variable*) and our explanatory variables of interest (*predictors* or *covariates*, *e.g.*, documentation need), under the effect of various *controls*.

### Model and variable selection

As our research questions are composed of three outcomes of interest (number of answers, answer speed, and answer quality), we require at least 3 models—one for each outcome. For examining the number of answers per question, we use a Poisson generalized linear model (GLM), as is standard with count data [46]. For examining the time to first good answer, we use ordinary least squares (OLS) regression with a logged dependent variable. Though time can be considered a count variable, we tested model fit between the OLS regression models and Poisson GLMs and found better fit with the OLS models. Finally, for answer quality models, we use logistic regression with a binary dependent label of “bad” or “good”, as discussed previously from the work by Ravi *et al.* [18].

In this work, all models except the model for answer quality are at the *question level*, *i.e.*, each observation is a question. For the answer quality model, each observation is an answer. As a result, for our time-to-answer models, we model the time to first *good answer*, where “good” is defined by answer quality label. We considered modeling at the *answer level* for all models; however, this would lead to multiple observations of the same question. Multiple observation can lead to high levels of correlation between covariates, potentially negatively affecting model inference. Among methods able to handle multiple observations are *mixed-effects* (or *random effects*) models. To test whether or not a mixed-effects model is necessary compared to a fully fixed-effects model (*i.e.*, if a random effect for question ID is necessary), we compare the *Akaike’s Information Criterion* (AIC) of the models with and without the corresponding random effect [47]. In the end, we decided against mixed-effects models both by their comparison of AIC and according to the principle of parsimony [48]; if the more complicated model is only marginally better, use the simpler model.

**Fig 2:**
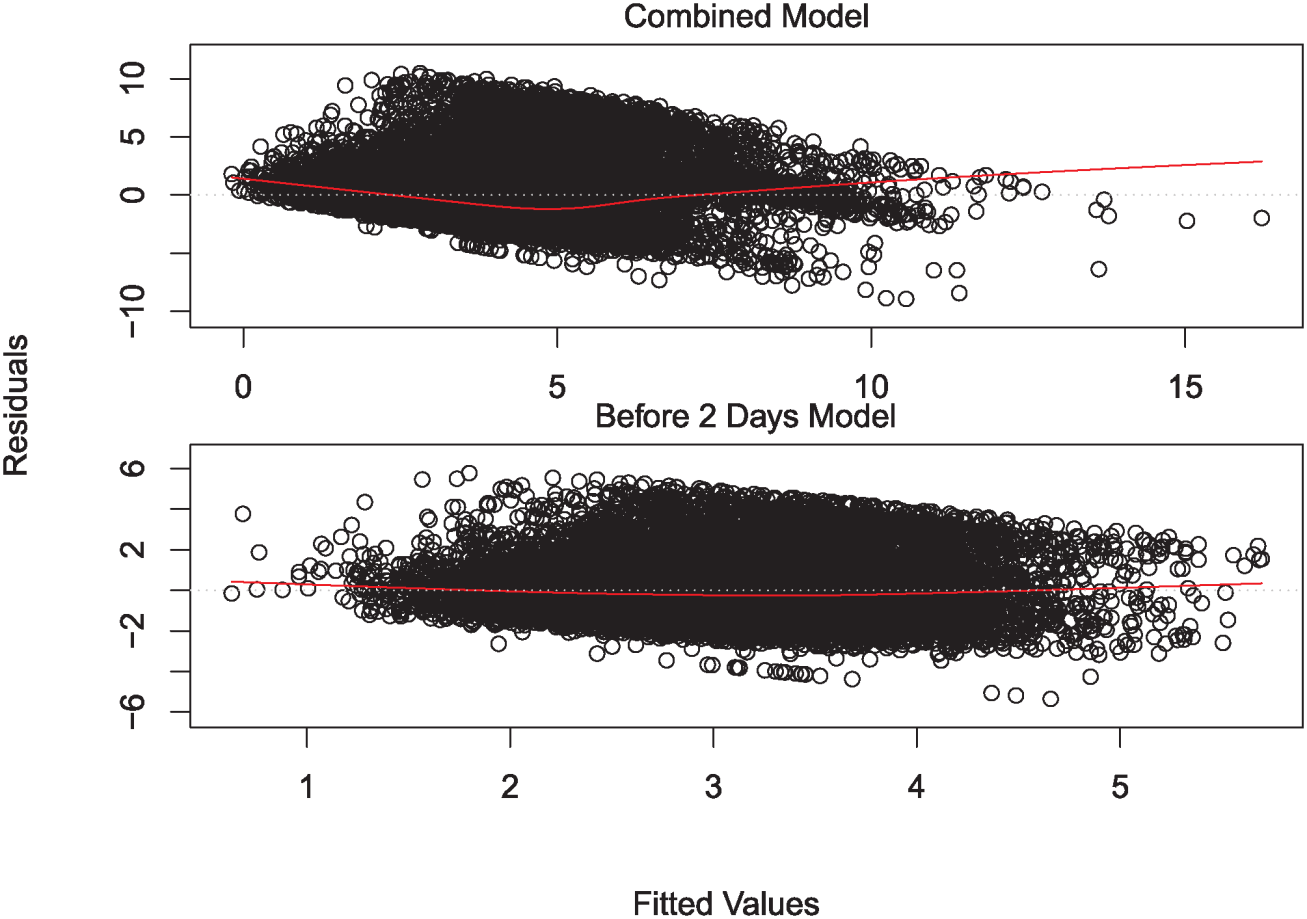
Residual vs. fitted value plots for combined and split time to answer models (lowess smoothed).

In addition, in order to observe the effect of the bounty on time to answer we separate our time to answer models into two parts: one for answers that come within 2 days of the question being asked, and one for answers after 2 days. This is because bounties can only be added 2 days after a question’s creation. Since most questions are answered within 2 days (88%), these questions necessarily cannot have a bounty, causing a very large skew in a combined model towards non-bountied questions. As a result, we believe that combining these two models would cause the bounty factor to be ineffective for inference, as the combined model is likely to be heavily biased towards non-bountied questions; in other words, the model will likely mostly capture the variance in non-bountied questions, as they are the vast majority of the data set. In fact, when examining the residuals vs. fitted values plot for the combined model, there is a comparatively poor fit for fitted values at and over 2 days. This can be seen in Fig 2; the combined model’s diagnostic plot has a comparatively large dip in the smooth line. Due to this poor fit for higher fitted values in the combined model, and the heavy skew towards non-bountied questions in the data, we separate the two models to make sure the bounty factor can be safely used for inference.

We employ *log* transformations on predictor variables to stabilize the variance and improve model fit when appropriate [49]. As explanatory variables are often highly correlated, we consider the *variance inflation factor* (VIF) of the set of predictors and compare against the recommended maximum of 5 to 10. All models presented have a maximum VIF of 3. To determine whether explanatory variables should be kept or removed, we compare models using likelihood ratio tests [50].

Variable names and descriptions can be found in Table 1. Note that some variables with a calculable answerer counterpart were computed, but not used in models due to issues of multicollinearity. In addition, some other variables were computed but not used due to issues of multicollinearity, *e.g.*, number of lines of code in the post body.

### Interpreting regression results

In ordinary least squares regression, R^2^ measures the percentage of variance captured by a model. However, a low R^2^ alone does not mean that the model cannot be inferred from [51–54]. We note relatively low R^2^ values in the time to answer models. The phenomenon we are modeling is a difficult one to fully capture—most questions are either answered very quickly, or reside in a very long tail; the range of values is large, but is heavily concentrated towards lower values. The differences between values within the heavy concentration is very small, and thus hard to model. We control for many factors that we believed may contribute in describing the variance in time to answer, guided by prior research. We also took great care to ensure that our models meet the assumptions of OLS regression by performing standard model diagnostics, and thus are still useful for inference, even if the R^2^ values may be considered low. It is important to note, however, that a low R^2^ increases the uncertainty in predicted values, even if p-values are low. For example, if one were to create a 95% confidence interval for estimates of significant coefficients in a model that has a low R^2^, the intervals will be larger when compared to a model with higher R^2^, even if the p-value of the coefficients are the same. As we don’t use our models for prediction, this drawback of using a low R^2^ model does not apply. However, it should be noted.

## Results

We begin our results by first examining a case study aimed at determining whether the new APIs in linked Stack Overflow questions are an integral part of the question, or if they are merely mentioned in passing. We then examine results for both our research questions.

### Case study: New API interest in Stack Overflow

One of our goals is to study the effect that newness of APIs mentioned in a question has on answer timeliness and quality. The implicit assumption is that question askers on Stack Overflow care about and use new APIs. For our models to be relevant, we must justify this assumption. In other words, we must make sure that our API linking strategy finds posts where the linked (new) API is an integral part of the discussion. Manually inspecting all questions linked to new APIs is infeasible, as we have 22,366 questions found by our linking strategy. Thus, we took a random sample of 50 questions linked to new APIs and manually categorized them as “explicitly about”, “involving”, or “not about” the linked new API. This categorization was performed independently by both authors. When combining the “explicitly about” and “involving” categories, this resulted in a 90% agreement rate; when keeping these groups separate, there was a 52% agreement rate. We note that although a 52% agreement rate may seem low, it is not unexpected. There are three categories, corresponding to a random agreement rate of 33%, and we argue that the task of identifying a question’s topic is difficult even for humans. Table 2 contains a coding guide that describes these defined categories.

**Table 2:** Qualitative coding guide for questions about Android APIs.

Code	Criteria
Explicitly About	1) The question directly references a particular API in the Android framework. 2) The API is the core component of the question; *i.e.*, confusion or curiosity regarding the API is explicitly stated, and the question entirely revolves around this particular API and, *e.g.*, its usage or idiosyncrasies.
Involving	1) The question directly references a particular API in the Android framework. 2) The API is explicitly a part of the question, but not necessarily the core component; *e.g.*, the referenced API is relevant and necessary to describe the question, but the core confusion or curiosity within the question revolves around something other than the referenced API, *e.g.*, a different API, or a general concern regarding the Android framework, not the referenced API specifically.

The “explicitly about” class is as its name: if the question is explicitly about the new API, it is classed as such. An example of such a question is:

*Question ID: 14620974*, *Linked API: SeekBar*

Title: Seekbar increase value up to 100

I have a seek bar with max = 25. What I want to do is when a user drags the seekbar to max value and it is in a pressed state […]

The “involving” class consists of questions that explicitly state the new API, but the question does not address it directly. An example of such a question is:

*Question ID: 11485026*, *Linked API: SeekBar*

Title: Seekbar creating EditTexts and then getting entries for further use

This code creates a seekbar and makes the seekbar create as many EditText fields as the slider is at / remove ones that would be too much. This code is in OnActivityCreated […]

As shown, the “involving” question above does explicitly mention the new API (*SeekBar*), but it is not clear that the question is entirely about the new API itself. We found that the difference between these two groups is often small, but still worth separating.

The results of our case study are as follows. Only 2 linked questions in the sample are not about the linked API. The two linked APIs in this case are *NetworkOnMainThreadException* and *ImageButton*. In the case of the former, the question is about the exception generated by the Android operating system itself, not the exception class. We avoid most of these issues with *Exception* classes due to our method of discovering APIs in questions, outlined in previous sections. For the latter, the question asker provided an incorrect user-defined tag.

**Fig 3:**
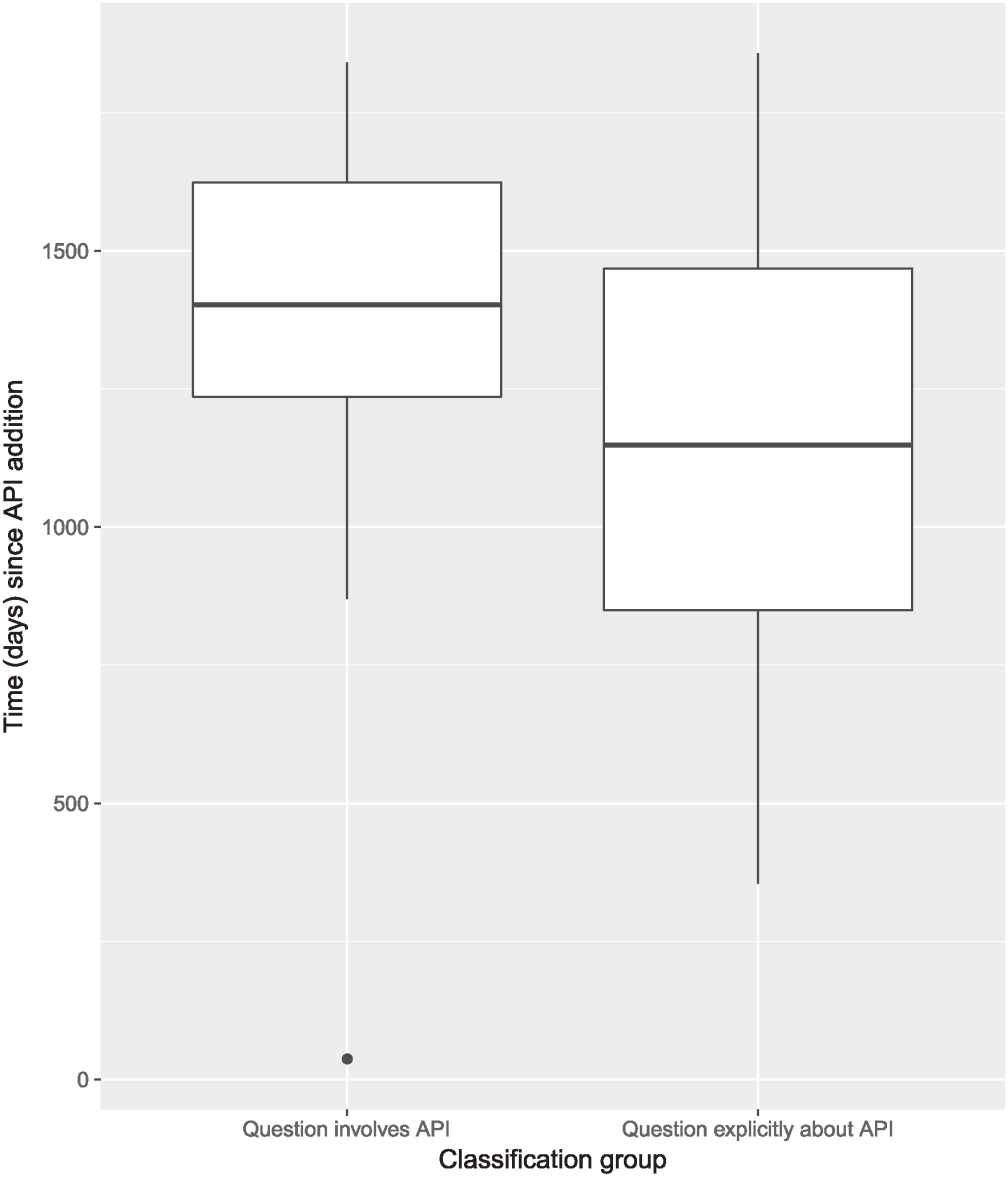
Time since API addition for 50 question case study, per manual classification group (2-sided t-test p < 0.05; 2-sided Wilcoxon rank-sum test p < 0.05).

As 48 of the 50 questions (22 “involving”, 26 “explicitly about”) in the case study are in either the “involving” or “explicitly about” groups, we have confidence that our models can be used for inference. To assuage potential concern about the difference between the “involving” and “explicitly about” groups, we sought to identify a control that can be used to separate between the two groups. Fig 3 shows time (days) since API addition for the new APIs referenced in the 50 case study questions per manually classified group. The box plot shows that questions in the “explicitly about” group are generally posed closer to the date of their referenced API’s addition than those questions in the “involving” group. This indicates that the number of days since the addition of a referenced API can be a useful control in dividing the “involving” from the “explicitly about” subgroups within the group of questions referencing new APIs. We use this control in all our models.

This case study shows that Stack Overflow users indeed ask questions about new APIs, and we can conclude that Stack Overflow users actually do use and care about new APIs; in other words, the new APIs are not merely mentioned in passing within the post. In addition, it provides confidence that our linking strategy indeed prioritizes true positive links, as false positives are rare. Meeting these core assumptions allows us to use the models we build for inference.

### Count, latency, and quality of answers to new API questions

#### Number of answers to new API questions

Table 3 shows our model for the number of answers per question. Column 1 serves as a base model; only controls for question creation date and question-related expertise metrics are used. Column 2 adds question-specific descriptive variables *i.e.*, textual variables and user-defined tag count. Column 3 introduces a variable that is not in control of the question asker (**QNComments**), API-related variables (**APIDiffTime, F.Added**), and variables related to the bounty (**F.Bounty, TimeToBounty**).

**Table 3:** Number of answers per question, Poisson GLM.

	Coefficient Estimates:		
	(1)	(2)	(3)
QCreationDate	−0.110***	−0.100***	−0.175***
QOwnerNQ	0.022***	0.016**	0.012*
QOwnerReputation	0.011**	0.012**	0.010*
QOwnerAge	−0.019***	−0.016***	−0.013***
QMEC	−0.015	−0.009	−0.006
QTitleLength		−0.056***	−0.033*
QBodyNWords		−0.048***	−0.054***
QBodyCharsOfCode		0.012***	0.007*
QNSwitches		−0.004	−0.014
QBodyURLCount		−0.018	−0.031**
F.QQualityLabelGood		0.083***	0.049***
QNeed		−0.018***	−0.014***
QNTags		−0.018***	−0.017***
QNComments			0.150***
F.Added			0.076***
APIDiffTime			−0.00005**
F.Bounty			0.203***
TimeToBounty			0.0003
Constant	1.305***	1.584***	2.075***
Log Likelihood	−32,400.610	−32,303.640	−31,589.460

*p<0.05

**p<0.01

***p<0.001

We see that high quality questions (**F.QQualityLabelGood**) receive more answers (0.049). For text-based, non-code variables (**QTitleLength, QBodyNWords, QBodyURLCount**), we see negative effects (−0.033, −0.054, −0.031). As these variables serve as proxies for textual complexity, this is expected. Stack Overflow emphasizes conciseness in question asking (https://stackoverflow.com/help/how-to-ask); as these variables represent question lengths, it is not surprising that longer questions receive fewer answers. We see that questions linked to new APIs (**F.Added**) receive more answers (0.076), when controlling for other relevant variables. This is a positive result for Stack Overflow—new APIs are a topic of interest to developers, and one would hope that their needs for the most current documentation is met. However, we see that questions with higher documentation need (**QNeed**) receive less answers (−0.014).

#### Latency of answers to new API questions

Tables 4 and 5 show our models for time to first good answer, for answers that come before and after 2 days. Column 1 serves as the base model. Column 2 adds answer-related variables, such as answerer expertise and answer text metrics. Column 3 adds question-related variables.

**Table 4:** Time to first good answer models, log minutes, before 2 days.

	Coefficient Estimates:		
	(1)	(2)	(3)
QCreationDate	−0.553***	−0.994***	−1.070***
QOwnerNQ	−0.184***	−0.158***	−0.110***
QOwnerReputation	0.050***	0.047***	0.012
QOwnerAge	0.044***	0.036***	0.039***
QMEC	0.249***	0.229***	0.192***
ABodyNWords		0.312***	0.262***
ABodyCharsOfCode		0.056***	0.055***
ANSwitches		−0.042**	−0.044**
ABodyURLCount		−0.008	−0.002
AMEC		−1.693***	−1.673***
AOwnerReputation		−0.139***	−0.135***
QNTags			0.071***
QTitleLength			0.098***
QBodyNWords			0.262***
QBodyCharsOfCode			0.009
QNSwitches			−0.030
QNComments			0.095***
QBodyURLCount			0.192***
F.QQualityLabelGood			0.181***
QNeed			0.045***
F.Added			0.133***
APIDiffTime			−0.0001**
Constant	7.035***	9.939***	8.994***
R^2^	0.032	0.133	0.164

*p<0.05

**p<0.01

***p<0.001

**Table 5:** Time to first good answer models, log minutes, after 2 days.

	Coefficient Estimates:		
	(1)	(2)	(3)
QCreationDate	−2.126***	−2.125***	−1.590***
QOwnerNQ	0.059*	0.062*	0.091***
QOwnerReputation	−0.068**	−0.023	−0.020
QOwnerAge	0.004	0.008	0.026
QMEC	−0.349***	−0.437***	−0.358***
ABodyNWords		−0.008	0.028
ABodyCharsOfCode		0.002	0.00001
ANSwitches		0.012	0.032
ABodyURLCount		0.113*	0.090*
AMEC		0.097	0.131
AOwnerReputation		−0.147***	−0.115***
QNTags			−0.060*
QTitleLength			−0.031
QBodyNWords			−0.093
QBodyCharsOfCode			−0.002
QNSwitches			−0.068
QNComments			0.032
QBodyURLCount			0.023
F.QQualityLabelGood			0.377***
QNeed			0.023
F.Added			−0.216*
APIDiffTime			0.0002**
F.Bounty			−1.240***
TimeToBounty			0.011***
Constant	26.115***	26.900***	23.176***
R^2^	0.157	0.184	0.291

*p<0.05

**p<0.01

***p<0.001

For questions with a first good answer within 2 days (Table 4), we see that questions linked to new APIs receive slower answers (0.133). For questions with a first good answer after 2 days (Table 5), we see that questions linked to new APIs receive faster answers (−0.216). When comparing these two models, the situation seems contradictory at first.

For the former case, the explanation could be that questions with new APIs are harder to answer, and thus answers come slower. This is supported by the fact that question text variables which serve as a proxy for complexity (*e.g.*, **QTitleLength**, **QBodyNWords**, **QBodyURLCount**) all have significant positive values. In addition, our data shows that there are far fewer unique people who answer questions that reference new APIs (2,266) than those who answer questions that are not about new APIs (7,264)—this may be due to new APIs requiring specific knowledge that is not yet widespread. Thus, due to a lack of knowledgeable individuals, answers come slower.

For the latter case, the explanation could be as follows. Questions with a first good answer after 2 days are harder to answer; otherwise, they would likely have received a faster answer (median time to first good answer in our data is 17 minutes). In addition, our data shows that of questions that are answered only after 2 days (2,525), only 46% are answered within 1 month. This result is more nuanced than what has been discussed in the past; questions that are hard enough to not receive an answer within 2 days often take longer than 1 month to answer—a far cry from the median answer time of 17 minutes. However, users want to document new APIs—this is supported by prior work that shows Android classes are highly documented, and generally done so quickly [3]. Thus, for questions that already take longer to answer (answers take ≥2 days to arrive), questions referencing new APIs receive comparatively faster answers (**F.Added** coefficient is −0.216).

For both time to answer models, we see that good quality questions take longer to answer. This is initially puzzling; if a question is of high quality, should it not be easier to answer? Recall that our metric for question quality is a function of question score. The mechanism that drives higher scores is complex and has been discussed in length by users (https://meta.stackexchange.com/q/130046/when-should-i-vote). However, the general consensus is that a question should be upvoted when it is useful, clear, and (or) shows research effort. It may be that an eminently useful question with no immediately obvious solution takes longer to answer. To our knowledge, there is no comprehensive (*e.g.*, qualitative) work on the social reasoning behind high Stack Overflow question scores. Thus, this explanation is one of a potential many; the underlying social mechanism behind high scoring questions could be the subject of future work.

For the first model (first good answer within 2 days), we see that questions with higher need receive slower answers (0.045). For the second model (first good answer within 2 days), **QNeed** is not significant, and is thus not considered.

#### Quality of answers to new API questions

Table 6 shows our model for answer quality. Column 1 serves as the base model. Column 2 adds answer-related variables, and column 3 adds question-related variables.

**Table 6:** Answer quality models (bad, good), logistic regression.

	Coefficient Estimates:		
	(1)	(2)	(3)
QCreationDate	−0.509***	−0.294***	−0.163***
TimeToAnswerMins	−0.095***	−0.064***	−0.092***
QOwnerNQ	−0.050***	−0.041***	−0.001
QOwnerReputation	0.203***	0.176***	0.136***
QOwnerAge	0.018***	0.024***	0.032***
QMEC	−0.114***	−0.074***	−0.068***
ABodyNWords		0.150***	0.142***
ABodyCharsOfCode		0.042***	0.048***
ANSwitches		0.105***	0.107***
ABodyURLCount		0.107***	0.075***
AMEC		1.679***	1.638***
AOwnerReputation		0.147***	0.138***
QNTags			0.020**
QTitleLength			−0.106***
QBodyNWords			−0.085***
QBodyCharsOfCode			−0.018***
QNSwitches			0.002
QNComments			−0.088***
QBodyURLCount			−0.030
F.QQualityLabelGood			0.764***
QNeed			0.032***
F.Added			−0.018
APIDiffTime			0.00003
F.Bounty			0.593***
TimeToBounty			0.001
Constant	2.638***	−1.350***	−1.429***
AUC	0.64	0.69	0.71

*p<0.1

**p<0.05

***p<0.01

We see a positive effect of question quality, indicating that higher quality questions receive higher quality answers, even when controlling for time to answer. This is in agreement with prior work [55]. We see no effect of new APIs on answer quality. However, we do see that documentation need has a positive effect on answer quality (0.032), indicating that APIs with high documentation need are more likely to receive a higher quality answer.

For significant text-based variables (**QTitleLength, QBodyNWords, QBodyCHarsOfCode**), we see negative effects. This suggests that decreased conciseness is associated with lower answer quality.

**Research Answer 1**: Questions referencing new APIs receive more answers. For questions with a first good answer within 2 days, questions referencing new APIs receive slower answers; for questions with a first good answer after 2 days, questions referencing new APIs receive faster answers. We see no significant effect of new APIs in identifying answer quality; however, APIs with higher documentation need are more likely to receive a higher quality answer.

### Count, latency, and quality of answers with a bounty

In regards to the bounty, we see net beneficial effects across the board. We see that questions with a bounty receive more (0.203, Table 3) and faster (−1.240, Table 5) answers, with higher quality (0.593, Table 6), all while controlling for the effects of new APIs.

**Fig 4:**
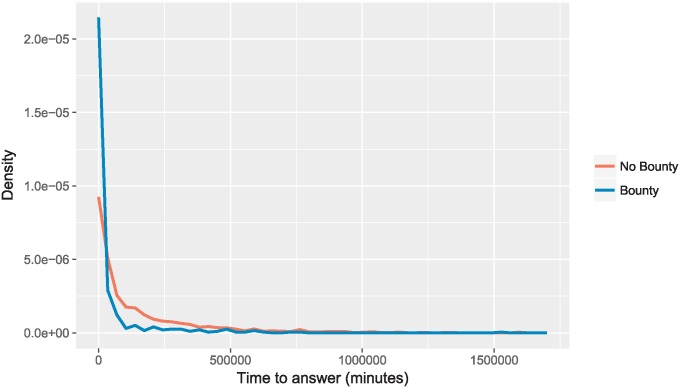
Time to answer density for non-bountied and bountied questions.

Looking further at our time to first good answer model (Table 5), we see that the bounty, with all other variables constant, decreases time to first good answer by a factor of *e*^−1.240^ = 0.289, *i.e.*, 71.1%. As shown in Fig 4, the bounty also has the effect of flattening the long tail of answer times, with a larger density towards smaller values of time. As discussed above, only 46% of questions without a good answer within 2 days are answered within 1 month; flattening of the long tail helps combat this issue. The stated goal of the bounty is to draw more attention to the bountied question—this is in hopes that the question asker will receive help due to the added attention. Here, we see that the bounty is effective in not only reducing the time to first good answer on average, but also in reducing the tail weight of the distribution of answer times. Thus, the bounty is a powerful tool in getting more, faster, and higher quality answers, even when controlling for the presence of new APIs (excluding answers which come before 2 days, where the bounty factor is undefined).

We also tested the inclusion of bounty amount into the models to see if higher reputation value bounties receive benefits compared to lower reputation value bounties (not shown in tables). In all forms individually tested (raw numeric 50, 100, *etc.*, scaled numeric 1, 2, *etc.*, factors for each raw value, and a binary factor of 50 *vs.* more than 50), there was no significant effect of bounty amount on any outcomes tested.

**Research Answer 2**: Questions with a bounty receive more, faster, and higher quality answers than those without a bounty. We find no significant effect of bounty amount on any outcomes tested, for any operationalization of bounty amount in each model.

## Discussion

Our results show that questions referencing new APIs receive more answers, when controlling for other relevant variables, a net positive for Stack Overflow that strives to provide timely, quality answers to questions. As stated by the Stack Overflow answering guidelines (https://stackoverflow.com/help/how-to-answer), users are instructed to *“make sure your answer provides [the specific answer for a given question]—or a viable alternative”*. In manual inspection of questions and answers for new APIs, answers often provide semi-orthogonal solutions to the same stated problem. When solving programming tasks, there are often many methods of accomplishing the same goal, and having multiple solutions provides a wider breadth of information and thus understanding as to how a given API works. We find the same is true in the case of the bounty; bounties increase the number of answers that a question receives, a beneficial effect. Thus, we conclude the bounty incentive works as intended in attracting more answers.

Our results also show that new API questions receive a slower first good answer, given that the answer comes within 2 days. This is a potential point of concern for Stack Overflow. Though the effect is relatively small in raw value (*e*^0.133^ = 1.142 or 14.2%), this may still be an issue if the question is asked, *e.g.*, close to the end of a work day, or if the solution is especially time critical. However, for new API questions which receive a first good answer after 2 days, we see a hastening effect; good news for Stack Overflow users.

In terms of documentation need, APIs in higher need receive higher quality answers (0.032), though slower answers for those questions answered within 2 days (0.045). In all, this is good news for Stack Overflow, as although answers may come slower for under-documented APIs, the answers they do receive are of higher quality.

In all cases, adding a bounty has a beneficial effect, increasing the number of answers, decreasing time to first good answer, and increasing answer quality. Interestingly, however, the bounty amount has no significant effect for any outcomes tested, for any operationalization of bounty amount tried. We acknowledge that the lack of a statistically significant operationalization does not necessarily equate to finding evidence that the bounty amount does not matter. However, we are still interested in discussing potential reasons behind this lack of significance, and do so below.

This lack of significance may elucidate an underlying phenomenon: Stack Overflow answerers may not care much about being “paid” extra for their work, as long as they are paid at all. Or, it could be that questions which receive a bounty are inherently more difficult to answer—those who answer these questions may be more expert and thus have more reputation to begin with, so the additional bounty payoff is negligible to them. Another explanation could be that the payment provides little to no motivation, and the increased benefits are due to the increased visibility the bounty provides. Further studies, including interviews and surveys of Stack Overflow users, are needed to distinguish among those alternatives.

If bounty visibility is the only reason that associated questions receive beneficial effects, one may consider replacing the bounty with, *e.g.*, a system that automatically detects underserved questions and randomly places them into a special section for a limited amount of time. However, Nisbett and Valins’ overly sufficient justification hypothesis [56] seems to argue against this; expected external incentives (here, the bounty reputation reward) can undermine intrinsic motivation for participation (*e.g.*, altruism), and when removed, can act against intrinsic motivation. On Stack Overflow, this means that removing the bounty and replacing it with an equivalent system that offers no reward may result in a negative response. Others have also discussed the bounty incentive in their work [4, 25, 32] and lauded its effectiveness. To our knowledge, we are the first to find that the bounty amount does not seem to matter for answer quality, speed, and amount, while controlling for many relevant variables.

Our findings suggest that in practice, one can put a bounty on their question and receive a large speed increase (71.1%), with the additional benefits of receiving more, higher quality answers. As we found the reputation reward for the bounty does not seem to matter, we believe putting up the minimal reward (50 reputation) is likely sufficient.

## Threats to validity

In addition to threats outlined in above sections, we acknowledge a number of threats to validity. First, our data is primarily from the Stack Overflow provided data dump. As mentioned, we found a number of issues in the provided data (*e.g.*, discrepancies when posts are migrated between sections within the Stack Exchange network). As we found these issues to affect a minority of our data (<1%), we do not believe this is a large threat. In addition, due to how the data dump is structured, it is expensive to calculate reputation scores at the time each question or answer is initially posted, as this requires calculating a cumulative sum for each user in our data across all their posts for all time. As we have 22,366 posts in our considered data, this calculation time is prohibitive. Thus, we use reputation scores at the time of the dump.

As shown in Fig 4, there is a long tail of answer times. We perform a split regression for our time to answer models for answers that come before and after 2 days; this may be seen as a threat. However, to properly study the effects of the bounty (which can only come 2 days after a question is asked), this split must be done, as it would be inappropriate to group questions which are ineligible to attain a bounty with those that are eligible. The fact that we do not further segment our regression beyond 2 days (*e.g.*, >2 days and <1 month) can also be seen as a threat. However, we log transform our answer time outcome variable in all models, which acts to reduce the effect of the long tail on model fit. As a result, we do not believe these threats are debilitating.

In this work, we identify an API as “new” if it has not been modified between the time of its introduction and the time the question is asked, for reasons described in Data. We acknowledge that this is only one of many choices for this definition. For example, we could define an API as new if it was added within some time *t* of a post being made, and test varying values of *t*. Or, we could define an API as new if it has only been added in the most recent framework update. Our choice has the drawback that some APIs may be defined as “new” by our method that are old in raw value; if an API was introduced in API level 1 and is not modified for all time, we would still consider this API as new. To mitigate this threat, we include various time variables (*e.g.*, **QCreationDate, APIDiffTime**) designed to absorb the variance introduced by these old (in raw time) APIs, and thus do not believe this threat affects our results significantly.

Though documentation metrics are split by API level and attached to posts based on time, we do not split function invocation counts by API level; they are aggregated across all levels. To split function invocation counts by API level would require far more data from the Google Play store, and may not be possible due to the fact that application developers often update their products to target newer APIs to, *e.g.*, remain up-to-date with the current trends [57, 58]. The Google Play store does not grant access to historical versions of applications. Thus, we cannot reliably gather enough data for function invocation counts per API level.

We note that our measure of “structural complexity” through counting HTML tag switches is not supported by prior work. We hypothesized its importance based on theories of language complexity used by, *e.g.*, Kincaid *et al.* [59]. Due to this initial hypothesis, we had to include it in our final models to retain inferential power (*i.e.*, we do not want to data peek; we do not want to remove a variable that we initially hypothesized as important after we found out it is not significant—this would be a disaster for inferential robustness). Though this may be seen as a threat, this variable was not significant in any of our models, and thus we do not interpret it in any discussions. It is possible that the inclusion of this variable may cause issues in estimation due to, *e.g.*, multicollinearity. However, we take extreme care to make our models robust for inference, including reducing multicollinearity as much as possible, as described in Methodology. As a result, we do not believe the inclusion of this variable negatively affects our findings.

## Conclusion

In this work, we studied Stack Overflow questions referencing new APIs, which we know have longer latency to an acceptable answer. Specifically, we sought to elucidate the factors affecting answer count, their latency, and their quality when the questions refer to new Android APIs.

Overall, we found that questions referencing new APIs receive more answers, after controlling for confounds, but there are subtleties in this overall result. Namely, among questions with a first good answer within 2 days, those referencing new APIs receive slower answers. On the other hand, among questions with a first good answer after 2 days, questions referencing new APIs receive faster answers. We see no significant effect of new APIs in identifying answer quality, but APIs with higher documentation need are more likely to receive a higher quality answer.

Questions with a bounty receive benefits across all variables of interest, though bounties can only be placed after 2 days. Based on these findings, we discussed what a question asker can do to receive benefits for our outcomes. In general, adding a bounty is the most efficient way to receive more, faster, and higher quality answers. However, the bounty reputation value does not appear to be significant in any context. We hypothesized potential reasons behind this insignificance. Due to the complexity of the bounty system and its introduction of many overlapping confounds, to our knowledge there is no prior work on the exact mechanism behind the bounty’s effectiveness, and why the amount is insignificant when controlling for confounds. This could be the subject of future work.

To our knowledge this is the first study that specifically focuses on questions referencing new APIs, and we use a novel metric to determine documentation need. These two points provide different descriptions of Stack Overflow’s ability to maintain currentness in terms of API documentation. In addition, we believe we are the first to find that the bounty amount does not seem to matter when controlling for many relevant confounds. We have identified both shortcomings and places where Stack Overflow excels in terms of maintaining currentness, and show that the bounty indeed accomplishes its intended effect. We hope our findings motivate others to identify and provide solutions for potential deficiencies in Stack Overflow, positively affecting software development as a whole.
